# Super-strong dislocation-structured high-carbon martensite steel

**DOI:** 10.1038/s41598-017-06971-w

**Published:** 2017-07-26

**Authors:** Jun-jie Sun, Yong-ning Liu, Yun-tian Zhu, Fu-liang Lian, Hong-ji Liu, Tao Jiang, Sheng-wu Guo, Wen-qing Liu, Xiao-bing Ren

**Affiliations:** 1 School of Materials Science and Engineering, State Key Laboratory for Mechanical Behavior of Materials, Xi’an Jiaotong University, Xi’an, 710049 P. R. China; 2 Frontier Institute of Science and Technology, Xi’an Jiaotong University, Xi’an, 710049 P. R. China; 3 Department of Materials Science and Engineering, North Carolina State University, Raleigh, NC 27695 USA; 4 School of Materials Science and Engineering, Nanjing University of Science and Technology, Nanjing, 210094 P. R. China; 5 Key Laboratory for Microstructure, Shanghai University, Shanghai, 200444 P. R. China; 6 Ferroic Physics Group, National Institute for Materials Science, Tsukuba, 305-0047 Ibaraki, Japan

## Abstract

High-carbon martensite steels (with C > 0.5 wt.%) are very hard but at the same time as brittle as glass in as-quenched or low-temperature-tempered state. Such extreme brittleness, originating from a twin microstructure, has rendered these steels almost useless in martensite state. Therefore, for more than a century it has been a common knowledge that high-carbon martensitic steels are intrinsically brittle and thus are not expected to find any application in harsh loading conditions. Here we report that these brittle steels can be transformed into super-strong ones exhibiting a combination of ultrahigh strength and significant toughness, through a simple grain-refinement treatment, which refines the grain size to ~4 μm. As a result, an ultra-high tensile strength of 2.4~2.6 GPa, a significant elongation of 4~10% and a good fracture toughness (K_1C_) of 23.5~29.6 MPa m^1/2^ were obtained in high-carbon martensitic steels with 0.61–0.65 wt.% C. These properties are comparable with those of “the king of super-high-strength steels”—maraging steels, but achieved at merely 1/30~1/50 of the price. The drastic enhancement in mechanical properties is found to arise from a transition from the conventional twin microstructure to a dislocation one by grain refinement. Our finding may provide a new route to manufacturing super-strong steels in a simple and economic way.

## Introduction

Materials with a combination of ultrahigh strength and high toughness are perpetually desired by human society^1, 2^, which are critical for energy efficient, lightweight structures and vehicles such as cars, airplanes and rockets. The “king” of such materials to date, is maraging steels, which are carbon-free but heavily alloyed steels containing a large fraction of expensive alloying elements including Ni, Co and Mo^3, 4^ (see details in Supplementary Table S1). High-grade maraging steel C350 exhibits an outstanding combination of super-high strength of 2.45 GPa, significant ductility of 6%, and good fracture toughness (K_1C_: 35~50 MPa m^1/2^)^3^. However, the high cost of maraging steels seriously limits their applications and inexpensive solutions for ultrahigh-strength and high-toughness steels are strongly desired. Over the past decades several notable inexpensive ultrahigh-strength steels based on interstitial carbon, the strongest strengthening but at the same time strongly embrittling element, have been developed, such as high-strength low alloying steels (HSLA)^3, 5^, and nano-bainite steel^6–9^. Although their strength has reached the level of medium-grade maraging steel C250, they are yet to achieve the strength level of high-grade maraging steel C350 with comparable ductility. Efforts in exploring ultrahigh-carbon (with C > 1.0 wt.%) steels as potential structural materials have led to some progress in non-martensitic steels and composites^10, 11^, but they are not comparable with super-high-strength steels. On the other hand, medium-strength non-martensitic steels can be work-hardened to possess ultrahigh-strengthen and significant ductility by various methods such as tempforming (TF)^12^, high-pressure torsion (HPT)^13^ and heavy cold-drawing (e.g., piano wires can exhibits a record-high strength of 6 GPa)^14, 15^; but the high-strength is restricted to small-dimensions like thin plate or wire. It is desired that an inexpensive ultrahigh-strength steel solution can be discovered not through processing the steels into small dimensions, so that a large degree of freedom in size and in shape can be endowed to the end products.

In this letter, we report a surprising finding that by a simple grain refinement treatment, glass-brittle high-carbon steels can be transformed into super-strong, monolithic steels with a combination of ultra-high strength and significant toughness, being comparable with that of high-grade maraging steel, but at merely 1/30~1/50 of the price. We further show that this remarkable change of the properties arises from a drastic change from twinned martensite to dislocation martensite.

## Results

**Figure 1:**
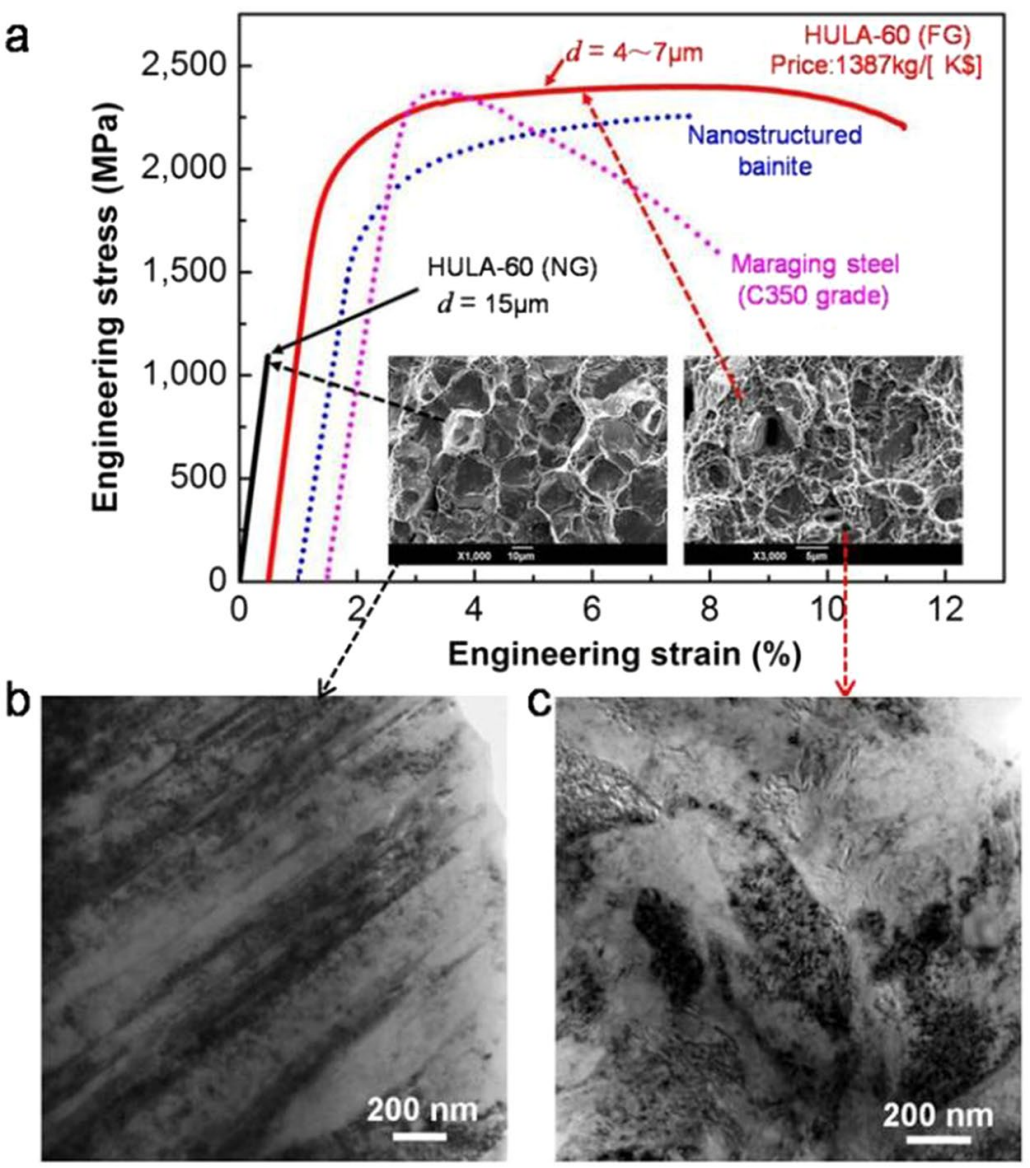
Comparison of mechanical properties of HULA-60 with other alloys as well as the grain size effect on the martensite substructures of HULA-60. (**a**) Engineering stress-strain curves of the NG (15 μm) and FG (4~7 μm) HULA-60 (see Supplementary Table S4 for details) samples as compared with a C350 grade maraging steels and a high silicon content nanostructured bainite steel^8^. (**b**) Twin-substructured martensite in NG HULA-60. (**c**) Dislocation-substructured martensite in FG HULA-60.

Figure 1a shows that a high-carbon ultrahigh-strength low-alloyed steel (HULA-60, see details in Supplementary Table S1) with composition (wt.%) 0.61C-1.5Cr-0.08Ni-0.05Ti-0.07Nb exhibits ultrahigh tensile strength (σ_0.2_ = 1.94 GP and σ_u_ = 2.4 GPa), high ductility (10%), and good fracture toughness (K_1C_: 29.6 MPa m^1/2^) when its grains were refined to 4~7 μm (fine grain or FG). It also exhibits extremely high compressive strength (over 7 GPa) and 70% strain without fracture (see details in Supplementary Table S2). The strength is comparable with that of C350-grade maraging steel but with higher ductility. Furthermore, the FG HULA-60 displays much better strain hardening ability than that of maraging steels, which is important for structure safety during overload. Compared with nano-bainite steels^8^, FG HULA-60 steel exhibits both higher tensile strength and higher ductility.

It should be noted that when having a normal grain (NG) size of 15 μm, the NG HULA-60 steel is still very brittle (Fig. 1a), in agreement with the common wisdom. Tempering at 500 °C can increase its fracture elongation to 12.1% but its strength drastically drops to 1289 MPa (see details in Supplementary Table S3). Therefore, the surprising combination of ultrahigh strength and good ductility cannot be achieved with normal grain size and is indeed associated with fine grains. To further confirm this, we designed another fine-grained steel with higher carbon content (0.65 wt.%), with a composition (wt.%) 0.65C-1.52Cr-0.09Ni-0.05Ti-0.07Nb. This sample, named FG HULA-65, exhibits even higher strength of 2.6 GPa, a significant ductility of 4% (for this strength level) and good fracture toughness (K_1C_: 23.5MPam^1/2^, see details in Supplementary Tables S1 and S2).

Figure 1b shows common twin substructure in the martensites of the NG HULA-60 sample, while Fig. 1c shows dislocation substructure in the martensite of the FG sample (see details in Supplementary Figure S1), which is unexpected for high carbon martensite. The fractography of twin martensite shows brittle intergranular fracture at prior austenite grain boundaries, whereas the dislocation martensite shows ductile fracture with many dimples, as shown in the insets of Fig. 1a.

**Figure 2:**
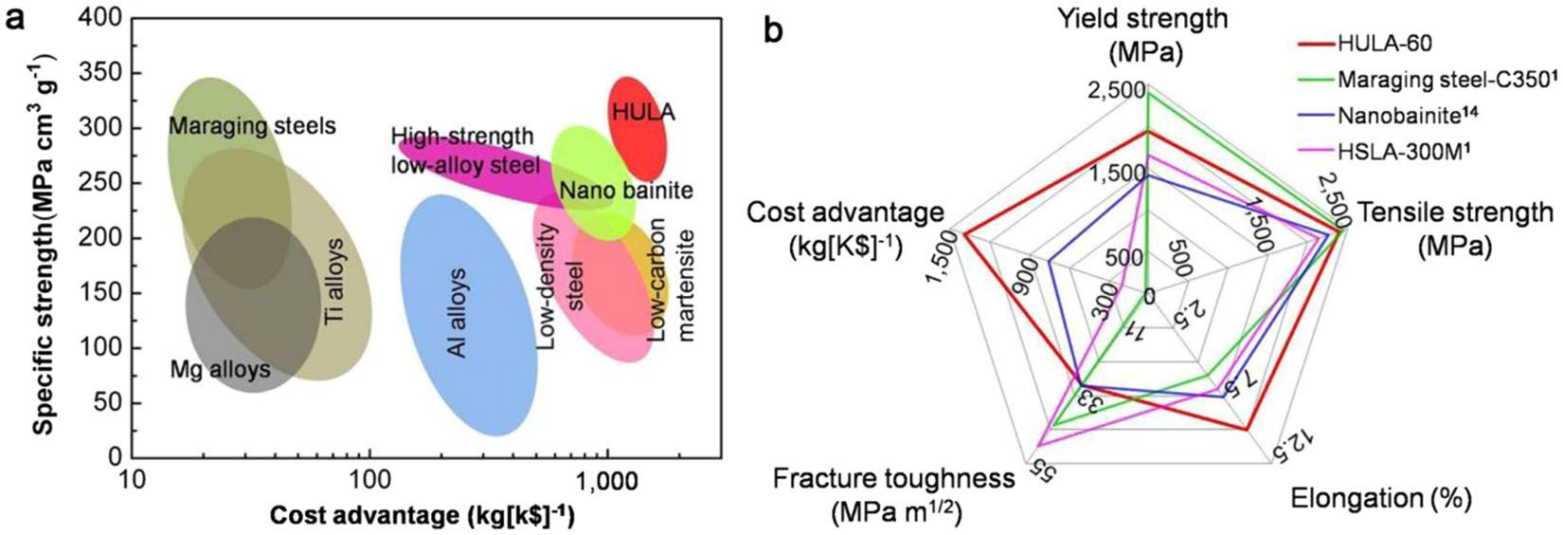
Comparison of mechanical properties and cost advantage of HULAs with other alloys and steels. (**a**) Specific strength versus the cost advantage (kg per thousand US dollars). (**b**) Comparison of tensile strength, yield strength, fracture toughness, elongation, and cost advantage for HULA-60 and several typical ultrahigh strength steels with yield strength exceeding that of low-grade Maraging steel (1700 MPa). The price of commercial materials is estimated from on-line quoted price and the price of non-commercial materials, such as HULAs and nanobainite, is estimated primarily based on the cost of alloying content.

Moreover, the super-strong HULA-60 is much cheaper than maraging steels. For example, it is estimated that the price of HULA-60 is only 1/50 that of C-350 (18Ni) grade maraging steel. Figure 2a shows that HULAs has the best combination of specific strength and price advantage as compared with known alloys. Figure 2b show a comparison of comprehensive properties (strength, ductility, toughness, and price advantage) of HULA-60 with several typical ultrahigh-strength steels such as C-350 (18Ni) maraging steel^3^, nanostructured bainite^9^ and HSLA 300 M^3^ (more details can be seen in Supplementary Figure S2). In this chart, materials strengthened by special deformation treatment to small dimensions (e.g., cold-drawing) are not included due to the aforementioned reason. The HULA-60 (red) shows excellent integrated mechanical properties and price advantage as compared with other ultrahigh strength steels.

**Figure 3:**
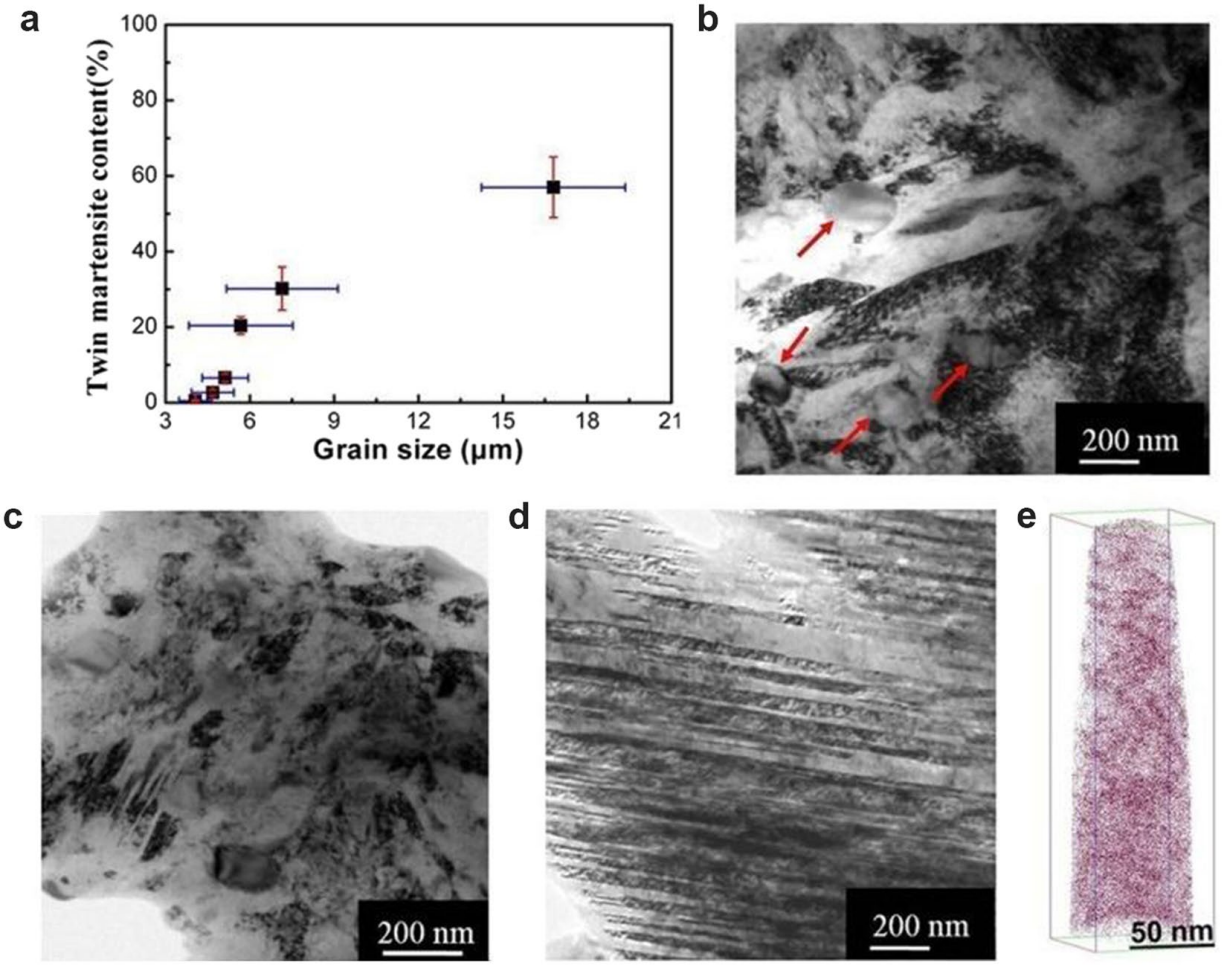
Grain size effect on martensitic substructure and carbon distribution in martensite matrix for an AISI52100 high carbon steel. (**a**) Influence of grain size on the fraction of twinned martensite. “Error bars” represent “standard deviations (s.d.)” (**b**) Full dislocation martensite at an average grain size of 4 μm. (**c**) A mixture of dislocation martensite and twinned martensite at an average grain size of 5.3 μm. (**d**) Fully twinned martensite at an average grain size of 16.8 μm. (**e**) APT-reconstructed 3D distribution of carbon atoms in the fine-grained martensite steel with an average grain size about 4 μm.

The sharp transition from a brittle high-carbon martensite to a super-strong and tough martensite seems to be associated with the observed grain-size-induced microstructure transition from twinned martensite to dislocation martensite, as shown in Fig. 1b and c. In order to confirm this finding, an AISI52100 steel with higher carbon content (with 0.98 wt.% C, see Supplentary Table S1) was studied because twinning is promoted by higher carbon content. Grains were refined in the same way as that of HULAs and the samples with varying grain sizes in the range of 4~16.8 μm were obtained by fine-tuning austenitizing temperature and time (details can be found in Supplementary Figure S3). Transmission electron microscopy (TEM) observations revealed that the fraction of twin substructure decreased with decreasing grain size (Fig. 3a), and a nearly full dislocation substructure appeared when the grains were reduce to about 4 μm, as shown in Fig. 3b (see also Supplementary Figure S4a), which also reveals some undissolved carbides (as marked by red arrows in Fig. 3b). With increasing grain size, twin martensite begins to appear at the grain size of 5.3 μm, where the microstructure is still dominated by dislocations (Fig. 3c and Supplementary Figure S4b). Twin substructure dominates at grain sizes of 16.8 μm and larger, which is the normal microstructure in high-carbon martensite steel (Fig. 3d). Moreover, atom probe tomography (APT)-reconstructed 3D distribution of carbon atoms (Fig. 3e) shows that about 0.78 wt.% C was detected in martensite matrix with fine grains of about 4 μm, which confirms that this martensite substructure transition is primarily caused by grain refinement, not by the content of the carbon dissolved in the austenite. The fine-grained AISI52100 samples with dislocation martensite exhibited some ductility after tempering at a low temperature, and ultrahigh strength (σ_0.2_ = 2.3 GP and σ_u_ = 2.4 GPa) with a tensile ductility of 0.4% was obtained when tempered at 280 °C, whereas the NG samples remained brittle even after being tempered at the higher temperature of 350 °C (see Supplementary Figure S5) because of its twin substructure^16, 17^. The lower ductility of FG AISI52100 is because of the large amount of undissolved carbides, which are known to reduce ductility by initiating cracks at carbide/matrix interfaces during deformation^18, 19^. This speculation is supported by the fact that ductility increased with increasing austenitizing temperature from 780 °C to 850 °C in HULA-60 (see Supplementary Table S3). Much higher ductility for HULA-60 treated at 850 °C is obtained because of less undissolved carbides (see Supplementary Figure S6). This is the major reason why the fine-grained AISI52100 displays lower ductility than HULA-60 and HULA-65.

## Discussion

It has been reported that refining grains can induce transition from twin shear to dislocation slip during the deformation of fcc, bcc and hcp metals^20–25^. Plastic deformation by slip and twinning are considered as competitive mechanisms^21^. For polycrystalline materials, it has been well-established that strength and grain size (*d*) follows a Hall–Petch law: *σ* = σ_0_ + k*d* ^−*α*^, where *σ* is yield stress, σ_0_ and k are constants and α is an exponent typically between 0.5 (for dislocation glide) and 1 (for twin)^21, 22, 24, 26^. The base of Hall–Petch law comes from dislocation glide, and it also has been used to fit the relationship of twin stress and grain size, however, the physical meaning of the constants σ_0_ and k are not clear. Usually, the Hall-Petch slope for deformation twinning is much larger than that for dislocation plasticity (listed in Supplementary Table S5)^21, 22, 24^, which means that the strength for twin deformation is much sensitive to grain size. Both the twinning stress and the slip stress increase with decreasing grain size, the onset of transition from twin shear to dislocation slip occurs when the twinning stress becomes equal to the slip stress^21^. Our observations indicate that refining grains can also induce martensitic substructure transition from twin to dislocations during the martensitic transformation of high carbon steel.

Here, we have presented a model based on twin and dislocation slip stresses to explain the transition mechanism from twin to dislocation with reducing grain size. Dislocation and twin are two kinds of substructures in martensite grains. They correspond to slip and twin deformations in martensitic transformation respectively. Suppose *τ*_*S*_ is slip critical shear stress and *τ*_*T*_ is twin critical shear stress, *γ*_*T*_ is twin shear strain. When twin and slip shear stress are equal, there will be a transition of deformation mechanism from twin to dislocation slip for high carbon martensitic transformation:

1 $${\tau }_{S}={\tau }_{T}$$

**Figure 4:**
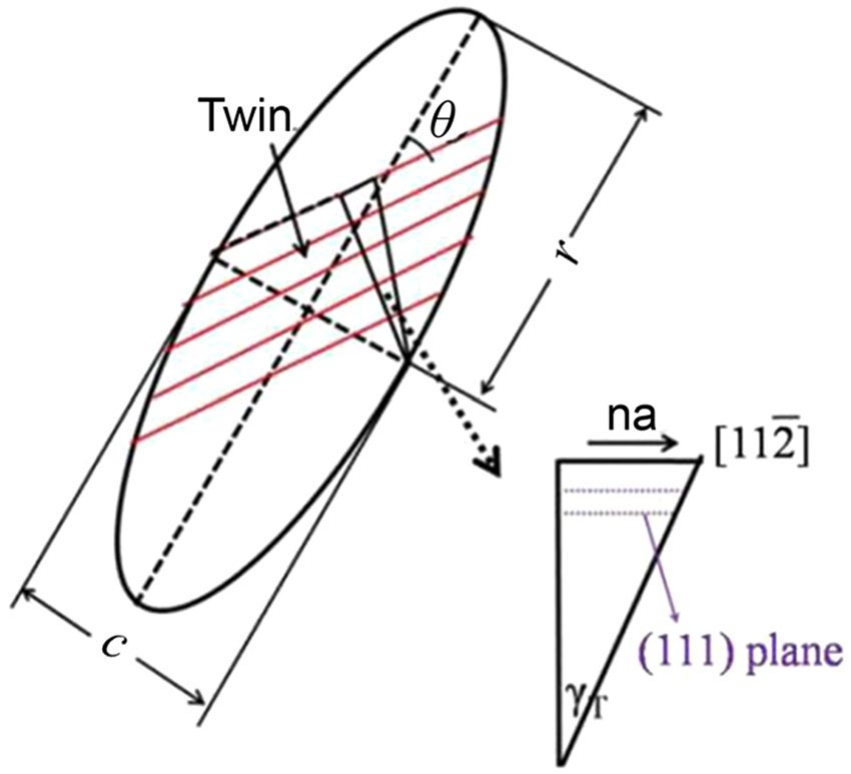
Schematic diagram showing the shear deformation of twin in a martensite plate.

As twin is a kind of shear deformation:

2 $${\tau }_{T}=G{\gamma }_{T}$$

where G is the shear Young’s modulus. Twin arises from uniform shear deformation of atoms in a region of crystal. From a schematic figure of twin deformation as illustrated in Fig. 4, the twin shear strain and the thickness *c* of a twin plate should be related by:

3 $${\gamma }_{T}=\frac{na}{c(\cos \,\theta )}$$

*a* is the distance between two adjacent atoms for a twin shear deformation in [11$\overline{2}$] direction of austenite, *θ* is the angle between twin direction and the martensite plate long axis, *n* is the number of interatomic distance in twin direction which describes the distance of displacement in twin deformation direction.

Suppose $\beta =\frac{c}{2r}$, a ratio of thickness to the length of the twin martensite plate, substituting Eq. (3) into Eq. (2) and letting *d* = 2*r*, the length of the first martensite plat is equal to grain diameter, we have:

4 $${\tau }_{T}=Gna{(\cos \theta )}^{-1}{\beta }^{-1}{d}^{-1}$$

The shear stress is proportional to *d* ^−1^, this is in good agreement with the measurement and analyzed result by Yu *et al*.^22^.

Meanwhile, the austenite yield stress will increase with reducing grain size, which makes the slip critical shear stress increase also. Generally, yield stress is related to grain size by:

5 $${\tau }_{S}={{\rm{\tau }}}_{{\rm{0S}}}+{\rm{k}}{d}^{-1/2}$$

where k is the Hall-Petch constant and τ_0S_ is a material constant representing slips resistance from lattice.

**Figure 5:**
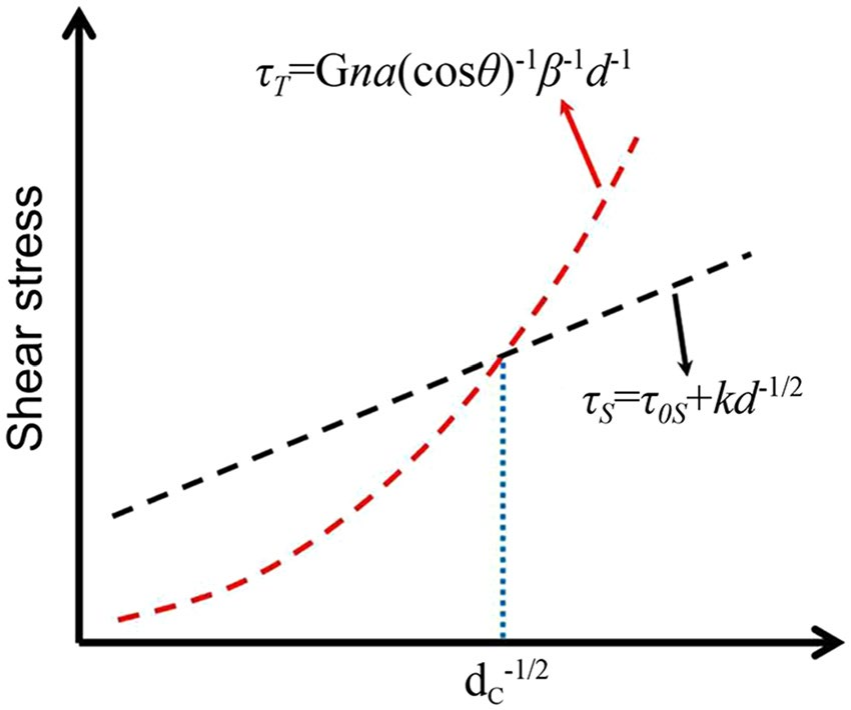
The schematic of relationship for slip shear stress and twin shear stress with grain size.

Both slip and twin shear stresses increase with reducing grain size, but twin shear stress increases faster as illustrated in Fig. 5. When the grain size is smaller than *d*_*c*_ (critical grain size), the slip shear stress is lower than the twin shear stress, i.e., dislocation-structured martensite is much easier to form.

In other words, whether martensitic transformation is facilitated by dislocation slip or deformation twinning depends on both the carbon content in the fcc austenite and the grain size. Decreasing grain size of austenite promotes dislocation slip and deters deformation twinning, which consequently changes the martensitic substructure from twins to dislocation cells. The dislocation structure enables martensite high ductility and toughness even when tempered at low temperatures, which produces a superior combination of strength and ductility. These findings provide some fundamental guidance for designing novel super-strong cheap steels. Furthermore, the simple thermomechanical treatment and heat treatment used in this experiment are compatible with existing processes of the steel industry and can be easily realized on conventional industrial production lines.

## Methods

### Preparation of the ultra-fine grained steels

The experimental HULA steels were melted in vacuum induction furnace and 10 kg ingot was casted with a diameter of 100 mm, and then were forged into square rods (70 × 52 mm^2^). And the commercial AISI52100 steel was also forged into square rods as that of HULA steels. After homogenization treatment at 1200 °C for 2 h, the square rods were hot-rolled in the temperature range between 950 °C and 850 °C with a total reduction of  65% and then cooled in air to room temperature. The initial fine grain size is achieved by a standard warm rolling^10, 27^ for multiple passes at 600 °C with a total reduction of  75%, and followed by annealing at 650 °C for 2 h and air cooling to room temperature. Finally, the ferrite grains are refined to less than 1 μm.

### Heat treatment for controlling austenite grain size before quenching

The UFG samples with dimensions of 10 × 10 × 5 mm^3^ were cut from the plates to explore the heat treatment parameters. The austenite grain size was controlled by fine-tuning austenitizing temperature and time during the austenitizing process after which the samples were quenched into water to room temperature. Hardness test was used to determine the time to complete austenitization at a given temperature. When the hardness of water-quenched specimen was higher than HRc 60, it indicated that the austenitization has been finished. Grain boundaries were revealed by an electrochemical etching method^28^, and the grain size was determined on the JX-2000 metallographic analysis software. And more than 500 grains were measured for each sample.

For AISI52100, Figure S3a in the Supplementary Information displays a series of experimental results of hardness and grain size variation with holding time at the same austenitizing temperature of 800 °C, and the minimum time needed to austenitize is about 2.5~3 min. Also, the variation of hardness and grain size with austenitizing temperatures at constant holding time of 3 min is shown in Supplementary Figure S3b, which shows that the hardness decreased slightly with increasing austenitizing temperature due to more retained austenite formation. Grains grow larger with longer holding time or higher temperature. Similar results are also observed in water quenched (WQ) HULA-60 and HULA-65 samples. From these experiments, the minimum grain sizes obtained are about 4 μm for AISI52100 and HULAs.

### Microstructure observation

TEM samples were sliced from these water-quenched AISI52100 and HULA-60 samples, and were mechanically polished to a thickness of about 30~40 μm followed by ion milling. Microstructure observation was carried out on a JEM-2100F transmission electron microscope.

A large number of observations confirm that the sub-microstructure of martensite is consisted of high density of dislocations in fine-grained (FG) AISI52100 (with grain sizes about 4 μm, heated at 800 °C for 3 min). More micrographs of microstructures are shown in the Supplementary Figure S4a, where no twin is observed. When the grains grew to 5.3 μm (heated at 850 °C for 3 min), the microstructure is still dominated by dislocation-substructured martensite (Supplementary Figure S4b) except for a small amount of twins. Another group of AISI52100 samples with varying grain sizes are obtained by varying holding time at 800 °C. When the steel is austenitized for 7 min, the mean grain size is about 4.7 μm, and twin begins to appear. As the holding time was prolonged to 30 min, grains grew to only about 5.8 μm; and the microstructure is still dominated by dislocation martensite. The volume percentage of twin martensite with varied grain size is estimated by Image-Pro Plus software on the TEM images, and the mean value is estimated from more than 20 images (with 10000× magnification) at each grain size value.

The microstructures of the water quenched tensile specimens before tempering of HULA-60 were observed with a field scanning electron microscope (SEM; JEOL JSM-6500F) as shown in Supplementary Figure S6. Specimens for SEM observation were prepared by mechanical polishing and etched with 3% nital.

### Carbon distribution reconstructed by APT

The specimen for atom probe tomography (APT) analysis was cut from the water quenched tensile specimen before tempering of AISI52100. The needle-like specimens for APT was prepared using a standard two stage electropolishing method at room temperature^29^. The APT experiment, using an advanced Imagolocal electrode atom probe (LEAP, 3000X HR, CAMECA, Gennevilliers, France), was performed at a residual pressure of 3 × 10^−9^ Pa and a specimen temperature of 50 K (−223 °C), and with a pulse repetition frequency of 200 kHz, a pulse-voltage to dc-voltage ratio of 15%. Image Visualization and Analysis Software (IVAS) was utilized for 3D reconstruction and composition analysis.

### Tensile and Charpy impact tests

Steel plates with the dimensions of 160 × 20 × 5 mm^3^ (length × width × thickness) were cut from the UFG plates, and the heat treatment was conducted as illustrated in Supplementary Table S4. After removing the possible decarburization layer, the tensile plate samples with gauge dimensions of 25 × 10 × 2.5 mm^3^ were prepared. The standard used for tensile testing is ISO 6892–1:2009. The tensile tests were conducted on Instron1195 electronic tensile testing machine at an extension rate of 0.2 mm/min. Steel plates with the dimensions of 180 × 15 × 5 mm^3^ (length × width × thickness) were processed in a same way as tensile specimen (see Supplementary Table S4). Then the processed plates were machined to V-notch Charpy impact specimens with thickness of 5 mm according to the standard of ISO 148–1:2009 and were also machined to plain-strain fracture toughness test specimens with sample size of 4.5 × 9 × 55 mm^3^ according to the standard of ISO 12737:2005. The Charpy impact tests were conducted on a MTS-ZBC3000 impact tester. The compression (with sample size of 3 × 3 × 4.7 mm^3^) and the fracture toughness tests were conducted on an Instron1195 testing machine.

## Electronic supplementary material

Supplementary Information
