# Supplementary material for: Super-strong dislocation-structured high-carbon martensite steel
Source: Sci Rep. 2017 Jul 26;7:6596. doi: 10.1038/s41598-017-06971-w (PMC5529530; doi:10.1038/s41598-017-06971-w)
Supplement: Supplementary file 1 — Supplementary Information [file 41598_2017_6971_MOESM1_ESM.pdf]

Supplementary Information for

Super-strong dislocation-structured high-carbon martensite steel

Jun-jie Sun[1,2], Yong-ning Liu*[1], Yun-tian Zhu*[3,4], Fu-liang Lian[1], Hong-ji Liu[1], Tao Jiang[1], Sheng-wu Guo[1], Wen-qing Liu[5], and Xiao-bing Ren*[2,1,6]

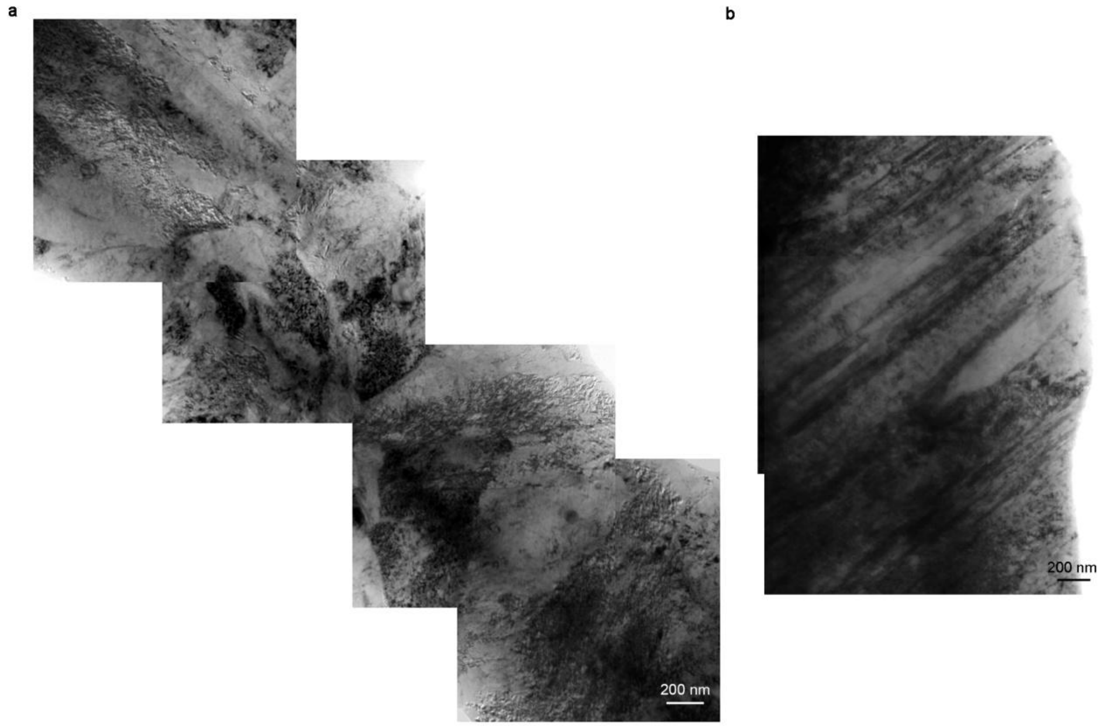

Supplementary Figure S1 | TEM microscopy of the martensitic substructure of HULA-60. a, Dislocation martensite in FG sample (4～7μm). **b**, Twinned martensite in NG sample (about 15μm).

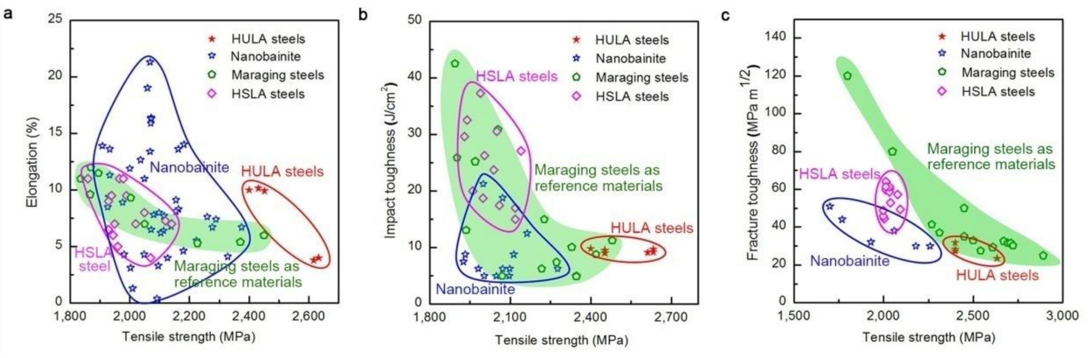

Supplementary Figure S2 | Comparison of comprehensive mechanical properties among nanobainite[6-9, 30-45], HSLA steels[3] and our HULA steels, with maraging steels[3, 46-55] as references (green region). a, ductility vs. strength. **b,** Impact toughness vs. strength. **c,** Fracture toughness vs. strength.

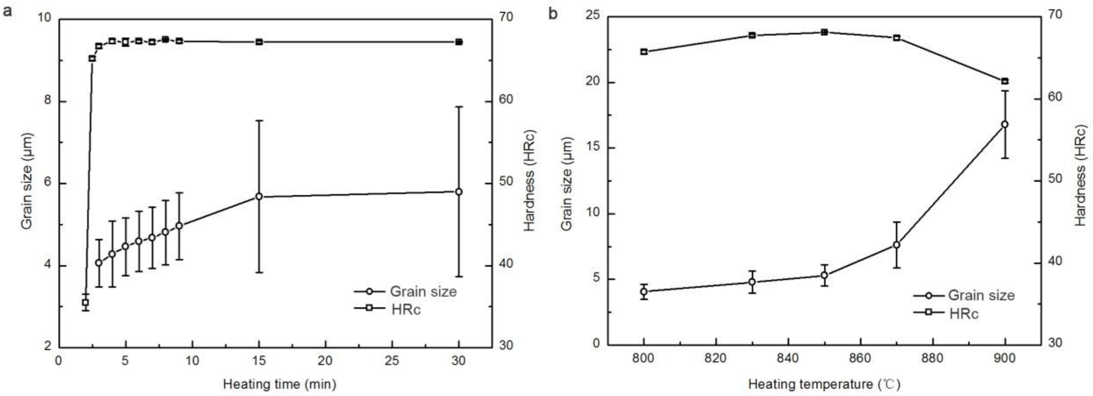

Supplementary Figure S3 | Variation of grain size and hardness of AISI52100 with heat treatment parameters, and "error bars" represent "standard deviations (s.d.)". a, Grain size and hardness change with varying holding time at a constant temperature of 800 ℃. **b**, Grain size and hardness change with varying heating temperatures at a constant holding time of 3min.

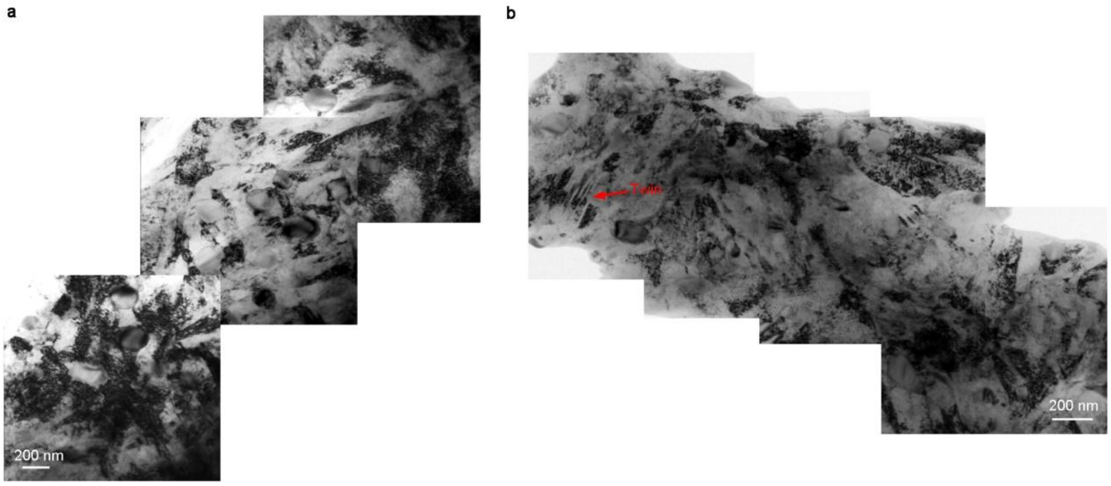

Supplementary Figure S4 | TEM microscopy of the martensitic substructure of AISI52100 at two different grain sizes. a, Dislocation martensite, at grain size of ~4μm (austenized at 800 ℃ for 3min). **b**, Primarily dislocation martensite mixed with a small fraction of twinned martensite, at grain size of about 5.3μm (austenized at 850 ℃ for 3min).

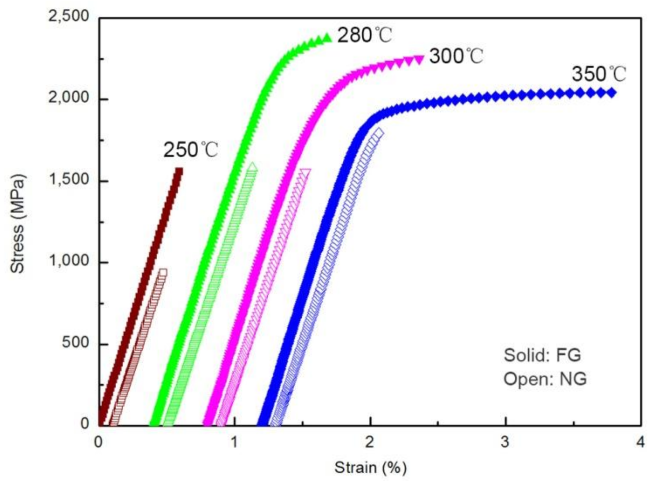

Supplementary Figure S5 | Contrasting mechanical properties of quenched and tempered AISI52100 steel between fine grained sample (FG) and normal grained sample (NG).

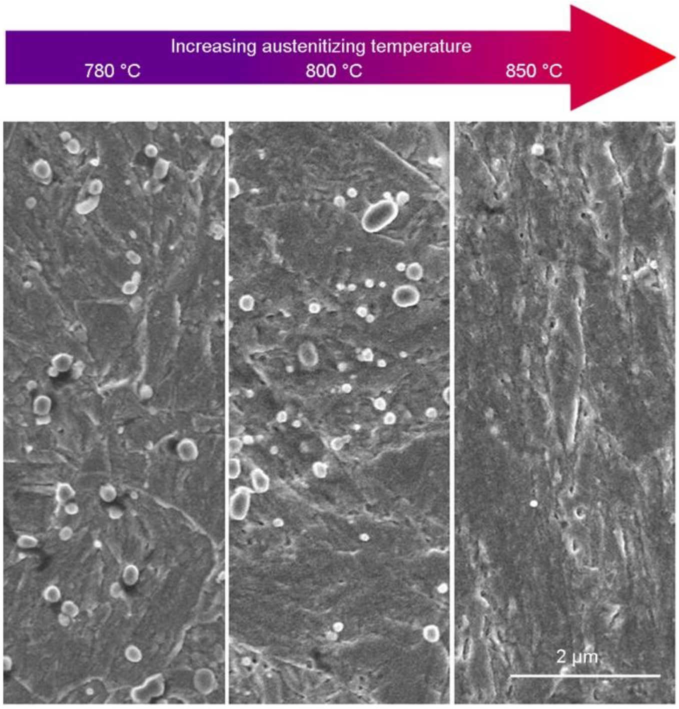

Supplementary Figure S6 | Vanishing undissolved carbides in HULA-60 with increasing austenizing temperature.

Supplementary Table S1丨Composition of the HULA steels and reference ultrahigh-strength steels.

Materials		Main alloying element content (wt.%)										
		C	Ni	Co	Cr	Mo	Ti	Nb	Al	V	Mn	Si
HULA	HULA-60	0.6	0.08	-	1.5	-	0.05	0.07	-	-	-	-
	HULA-65	0.65	0.09	-	1.52	-	0.05	0.07	-	-	-	-
Maraging steel [3]	18Ni (C250)	0.01	18	8.5	-	5	0.4	-	0.1	-	-	-
	18Ni (C300)	0.02	18	9	-	5	0.7	-	0.1	-	-	-
	18Ni (C350)	0.007	18	12	-	4.7	1.4	-	0.13	-	-	-
Nano-bainite [6, 8, 9]	Alloy-1	0.98	0.16	-	0.45	-	-	-	-	-	0.77	2.9
	Alloy-2	0.6	-	-	1.29	0.25	-	-	-	0.1	1.99	1.6
	Alloy-3	0.79	-	-	1.01	0.25	-	-	-	0.1	1.0	1.55
	Alloy-4	0.79	-	1.51	1.01	0.24	-	-	1.01	-	1.98	1.56
HSLA [3]	AISI4140	0.4	-	-	1.0	0.2	-	-	-	-	0.85	0.3
	AISI4340	0.4	1.8	-	0.8	0.25	-	-	-	-	0.7	0.3
	300M	0.43	1.8	-	0.85	0.4	-	-	-	-	0.8	1.6
	AISI6150	0.5	-	-	1.0	-	-	-	-	0.2	0.8	0.3
AISI52100		1.0	0.08	-	1.5	0.01	-	-	-	-	0.34	0.2

Supplementary Table S2 | Comparison of mechanical properties among HULA steels, maraging steels, nano-bainite steels, and HSLA (high-strength low alloy) steels.

Materials		YS (MPa)	UTS (MPa)	Elongation (%)	Fracture toughness (MPa m$^{1/2}$)	Toughness (J/cm^2)	Comment	Ref.
HULA	HULA-60	1943	2400	10	29.6	24.5*	Tensile	
		2122	7232	68.4	-	9.8†	Compression	
	HULA-65	2189	2633	4	23.5	23*	Tensile	
		2403	7485	67.5	-	9.2†	Compression	
Maraging steel	18Ni (250)	1700	1800	8	120	46.25†	Tensile	3, 54, 55
	18Ni (300)	2000	2100	5.62	-	24.95†		
	18Ni (350)	2400	2450	6	35-50	15†		
Nano-bainite	Alloy-1	1704	2287	7.4	-	6.25†	Tensile	6, 8, 9
	Alloy-2	1669	2048	4.3	-	5†		
	Alloy-3	1673	2098	8	-	6.25†		
	Alloy-4	1410	2260	7.63	30	-		
HSLA	AISI4140	1740	1965	11	-	18.75‡	Tensile	3
	AISI4340	1860	1980	11	44.5-61.5	25‡		
	300M	1650	2140	7	49.3-57.4	27.15‡		
	AISI6150	1810	2050	1	-	-		

*Charpy U-notch impact toughness; †Charpy V-notch impact toughness. ‡Izod impact toughness.

Supplementary Table S3 | Mechanical properties of the HULA-60 at different heat treatment parameters.

Heat treatment	YS (MPa)	UTS (MPa)	Elongation (%)
780°C quenching, 200°C tempering (FG)	1968	2234	6.8
800°C quenching, 200°C tempering (FG)	1936	2236	7.5
850°C quenching, 200°C tempering (FG)	1943	2400	10
850°C quenching, 500°C tempering (NG)	1152	1289	12.1

Supplementary Table S4 | Heat treatment summary of the tensile and Charpy impact specimens.

Materials	Austenitizing process	Cooling method	Tempering process	Grain size
AISI52100 (FG)	Heating at 800°C for 8min	WQ	250°C, 280°C, 300°C and 350°C for 1.5h respectively	About 4μm
AISI52100 (NG)	Heating at 900°C for 8min	OQ	250°C, 280°C, 300°C and 350°C for 1.5h respectively	About 15μm
HULA-60 (FG)	Heating at 700°C ,800°C and 850°C for 8min respectively	WQ	200°C for 1.5h	4～7μm
HULA-60 (NG)	Heating at 850°C for 15min or heating at 900°C for 8min	WQ	200°C, 500°C for 1.5h	About 15μm
HULA-65 (FG)	Heating at 860°C for 10min	WQ	250°C for 1.5h	4～6μm

WQ, water quenching; OQ, oil quenching.

Supplementary Table S5 | Comparison of Hall-Petch slopes for slip and twinning[21,22,24].

Materials	H-P slope for dislocation slip (MPa μm$^{1/2}$)	H-P slope for twinning (MPa μm$^{1/2}$)
BCC		
Fe-3wt.%Si (Hull)	328.88 (RT)	1216.85
	557.83 (77K)	
Fe-3wt.%Si (Loehe and Vöhringer)	379.48	3162.3
Armco Fe (Loehe and Vöhringer)	632.46	3921.25
Armco Fe (moiseev and Trefilov)		2846.1
Steels: 1010, 1020, 1035 (Loehe and Vöhringer)	632.46	3921.25
Fe-25 at.% Ni (BCC) (Nilles and Owen)	1043.56	3162.3
Cr (Marcinskowski and Lipsitt)	318.76	2142.46
FCC		
Cu (Vöhringer)		683.06 (77K)
(Meyers et al.)	170.76 (RT)	
(Zerilli and Armstrong)	164.44 (RT)	
Cu-6 wt.% Sn	224.52	373.15 (77K)
Cu-9 wt.% Sn	259.31	249.82 (77K)
Cu-10 wt.% Zn	224.52	496.48 (77K)
Cu-15 wt.% Zn (Vöhringer; Koester and Speidel)	265.63	373.15 (77K)
HCP		
Zr (Song and Gray)	260.89	2504.54
Ti (Okazaki and Conrad)	189.74 (78K)	569.21 (4K)
Mg-3Al-1Zn	297.26 (298K)	
	303.58 (373K)	
	294.1 (423K)	3099.05 (423K)
	271.96 (473K)	2719.58 (473K)

References

30 Rementeria, R. *et al.* On the role of microstructure in governing the fatigue behaviour of nanostructured bainitic steels. *Mater. Sci. Eng.,A* **630**, 71-77 (2015).

31 Caballero, F. G., Garcia-Mateo, C. & Miller, M. K. Design of novel bainitic steels: moving from ultrafine to nanoscale structures. *JOM* **66**, 747-755 (2014).

32 García-Mateo, C. & Caballero, F. G. The role of retained austenite on tensile properties of steels with bainitic microstructures. *Mater. Trans.* **46**, 1839-1846 (2005).

33 Bhadeshia, H. K. D. H. In: Proceedings of the 1st International Symposium on Steel Science (IS3–2007), *ISIJ*, (2007).

34 Bhadeshia, H. K. D. H. Properties of fine-grained steels generated by displacive transformation. *Mater. Sci. Eng., A* **481**, 36-39 (2008).

35 Bhadeshia, H. K. D. H. The first bulk nanostructured metal. *Sci. Technol. Adv. Mat.* **14**, 100-104 (2013).

36 Yoozbashi, M. & Yazdani, S. Mechanical properties of nanostructured, low temperature bainitic steel designed using a thermodynamic model. *Mater. Sci. Eng., A* **527**, 3200-3205 (2010).

37 Yoozbashi, M., Yazdani, S. & Wang, T. Design of a new nanostructured, high-Si bainitic steel with lower cost production. *Mater. Des.* **32**, 3248-3253, (2011).

38 Avishan, B., Yazdani, S. & Nedjad, S. H. Toughness variations in nanostructured bainitic steels. *Mater. Sci. Eng., A* **548**, 106-111 (2012).

39 Avishan, B., Garcia-Mateo, C., Morales-Rivas, L., Yazdani, S. & Caballero, F. G. Strengthening and mechanical stability mechanisms in nanostructured bainite. *J. Mater. Sci.* **48**, 6121-6132 (2013).

40 Garcia-Mateo, C. *et al.* Nanostructured steel industrialisation: plausible reality. *Mater. Sci. Technol.* **30**, 1071-1078 (2014).

41 He, J., Zhao, A., Zhi, C. & Fan, H. Acceleration of nanobainite transformation by multi-step ausforming process. *Scripta Mater.* **107**, 71-74 (2015).

42 He, J., Zhao, A., Huang, Y., Zhi, C. & Zhao, F. Acceleration of bainite transformation at low temperature by warm rolling process. *Mater. Today: Proceedings* **2**, S289-S294 (2015)

43 Garbarz, B., Marcisz, J., Burian, W. Technological peculiarities of manufacturing nanobainitic steel plates. *Metec Congress*, 209-210 (2015).

44 Yang, J., Wang, T., Zhang, B. & Zhang, F. Microstructure and mechanical properties of high-carbon Si–Al-rich steel by low-temperature austempering. *Mater. Des.* **35**, 170-174 (2012).

45 Yang, J., Wang, T., Zhang, B. & Zhang, F. High-cycle bending fatigue behaviour of nanostructured bainitic steel. *Scripta Mater.* **66**, 363-366 (2012).

46 Viswanathan, U., Dey, G. & Asundi, M. Precipitation hardening in 350 grade maraging steel. *Metall. Mater. Trans. A* **24**, 2429-2442 (1993).

47 He, Y., Yang, K. & Sha, W. Microstructure and mechanical properties of a

2000 MPa grade Co-free maraging steel. *Metall. Mater. Trans. A* **36**, 2273-2287 (2005).

48 He, Y., Yang, K., Sha, W. & Cleland, D. Microstructure and mechanical properties of a 2000 MPa Co-free maraging steel after aging at 753 K. *Metall. Mater. Trans. A* **35**, 2747-2755 (2004).

49 Ahmed, M. *et al.* Reclamation and additional alloying of 18Ni (350) maraging steel. *J. Mater. Eng. Perform.* **3**, 386-392 (1994).

50 Shetty, K., Kumar, S. & Rao, P. R. Effect of ion nitriding on the microstructure and properties of Maraging steel (250 Grade). *Surf. Coat. Technol.* **203**, 1530-1536 (2009).

51 Kempen, K., Yasa, E., Thijs, L., Kruth, J.-P. & Van Humbeeck, J. Microstructure and mechanical properties of Selective Laser Melted 18Ni-300 steel. *Physics Procedia* **12**, 255-263 (2011).

52 Ahmed, M., Nasim, I. & Husain, S. Influence of nickel and molybdenum on the phase stability and mechanical properties of maraging steels. *J. Mater. Eng. Perform.* **3**, 248-254 (1994).

53 Ooi, S. W., Hill, P., Rawson, M. & Bhadeshia, H. K. D. H. Effect of retained austenite and high temperature Laves phase on the work hardening of an experimental maraging steel. *Mater. Sci. Eng., A* **564**, 485-492 (2013).

54 Viswanathan, U., Dey, G. & Sethumadhavan, V. Effects of austenite reversion during overageing on the mechanical properties of 18 Ni (350) maraging steel. *Mater. Sci. Eng., A* **398**, 367-372 (2005).

55 Hoseini, S. R. E., Arabi, H. & Razavizadeh, H. Improvement in mechanical properties of C300 maraging steel by application of VAR process. *Vacuum* **82**, 521-528 (2008).
